# Accidental blood exposure: risk and prevention in interventional radiology

**DOI:** 10.2349/biij.2.4.e55

**Published:** 2006-10-01

**Authors:** A Vijayananthan, LH Tan, A Owen, R Bhat, R Edwards, I Robertson, JG Moss, R Nicholls

**Affiliations:** 1 Department of Biomedical Imaging (Radiology), Faculty of Medicine, University of Malaya, Kuala Lumpur, Malaysia; 2 Department of Medicine, Faculty of Medicine, University of Malaya, Kuala Lumpur, Malaysia; 3 Department of Radiology, Gartnavel General Hospital, Glasgow, United Kingdom

**Keywords:** Interventional radiology, bloodborne pathogens, prevention

## Abstract

There is a growing concern about the transmission of bloodborne pathogens during medical procedures among health care workers and patients. Over the last three decades, radiological services have undergone many changes with the introduction of new modalities. One of these new disciplines is interventional radiology (IR) which deals with procedures such as arteriography, image-guided biopsies, intravascular catheter insertions, angioplasty and stent placements. Despite these developments, the potential for accidental blood exposure and exposure to other infectious material continues to exist. Therefore, it is important for all radiologists who perform invasive procedures to observe specific recommendations for infection control. In this review, we look at the different policies for protection and universal standards on infection control.

## INTRODUCTION

Blood borne pathogens are a definite threat to health care workers due to potential occupational exposure. Concern about Hepatitis B (HBV), Hepatitis C (HCV) and the Human Immunodeficiency Virus (HIV) are rising in societies globally. This is especially true for interventional radiologists who perform diagnostic and therapeutic procedures involving needles and catheters. Many practitioners have expressed the need for official statements regarding the issue of infection control and prevention unique to interventional radiology. The Society of Cardiovascular and Interventional Radiology (SCVIR) Subcommittee on HIV and Bloodborne Pathogens was formed to review current knowledge on the risk of transmission during interventional procedures, to summarize regulations and formulate prevention policies [1]. The Guidelines for Prevention of Intravascular Catheter-Related Infections was developed by the Center for Disease Control and Prevention in 1996 and updated in 2002. These were developed for practitioners who insert catheters and health care workers responsible for the surveillance and control of infection in the hospital [2,3]. Studies have also been carried out involving the risk of infection to interventional radiologists. The message from these studies is that the risk of infection to interventional radiologists is real and the standards of infection control are suboptimal.

## RISK OF PATHOGEN TRANSMISSION

There is a wide disparity in the figures regarding the prevalence of infection in developed and developing nations. A number of factors influence the risk of pathogen transmission. The nature of potentially infectious material, the likelihood of sharps or other exposures occurring during the procedure, the prevalence of infection in the population, the seroconversion rates after exposure and the type of procedure undertaken, are some of the important criteria that need to be considered [1].

An estimated two billion people have been infected by HBV worldwide and there are approximately 50 million new cases diagnosed annually. Seventy five percent of such cases are found in Asia [4]. The evidence of HBV exposure in the general population in the United States is approximately 3-14% with an estimated 0.1-0.7% of the general population being HBV carriers [1]. Asian statistics showed a prevalence of hepatitis B markers in the general population in the Philippines and Malaysia to be 5-16% and 3-5% respectively [4].

Approximately three percent of the world population is chronically infected with HCV [5]. In France, the prevalence of HCV in the general population is estimated to be about 1.05-1.2% while in the United States it is almost 2% and Malaysia carries a figure of 0.6-1.0% [4,6]. In the Philippines the prevalence of HCV in the general population is estimated to be about 5.2%. Approximately 15-20% of patients with HCV will develop cirrhosis [4]. Unlike HBV, there is no vaccine for the prevention of HCV and HCV-related end stage liver disease is the most common indication for liver transplant in adults in the Unites States [7].

Globally, an estimated 40 million people are currently living with HIV, and about 20 million people have already succumbed to it, with the worst of the epidemic centred in Africa [8]. As of 2005, there has been an estimated five million new cases of HIV and the virus shows no sign of abating. The prevalence of HIV in the general population in the United States is estimated to be less than one percent. Studies at an urban trauma center found that 0.2-14.2% of inpatients at the acute care wards, and six percent of unselected patients observed in the emergency department of the same center were HIV positive [9].

HIV and HBV are found in most body fluids like blood, breast milk, cerebrospinal fluid, semen, vaginal secretions, pleural, pericardial, peritoneal or amniotic fluid and saliva. Faeces do not contain these viruses unless contaminated with blood. Urine is also not considered to be infective unless the patient has sustained trauma or undergone instrumentation to cause bleeding [1,9]. HIV survives on environmental surfaces at room temperature for a very brief period, but HBV can survive for about seven days. Three phases of chronic HBV infection are recognized. In Phase 1, the patients are HbeAg positive with high serum levels of virus and minimal hepatitis. In Phase 2, there is intermittent or continuous hepatitis and in Phase three (inactive phase), the viral concentrations are at its lowest and there is minimal liver involvement [4].

The risk of needle stick or other sharps injuries during an interventional radiology procedure is quite low when compared to the risk in surgical procedures. In a prospective study of 501 procedures, Hansen *et al.* found an injury rate of only 0.6% [10]. In a national survey done in the United States, the median number of injuries per year was found to be considerably low at 0.3 for practicing interventional radiologists [9].

Percutaneous exposure poses a greater risk as compared to mucocutaneous exposure. In IR procedures, cutaneous exposure is more common due to the frequency of glove perforations and splash-type exposures. Occult glove perforations were found in 23% of gloves worn for more than two hours and cutaneous blood exposure occurred in 3% [1,9]. Studies in the United Kingdom by Davidson *et al.* and McWilliams *et al.* stated that splashing and spraying of blood occurred in 6.7-8.7% of angiographic procedures [11,12]. The risk of accidental blood exposure was also found to be greater for procedures requiring more than two catheter exchanges, for thrombolysis and angioplasty as well as for procedures lasting more than 30 min [9].

For a given exposure, seroconversion depends on several factors, including the type of exposure (percutaneous, cutaneous or mucous membrane), severity, type of body fluid involved and the patients’ immune status [1]. The type of instrument is also important in sharps injuries as large bore needles have a greater volume of infected material. Seroconversion of blood borne pathogens has been reported after percutaneous exposure and contact with mucous membranes and non-intact skin, but not after contact with intact skin [9].

According to past data, the risk of HIV transmission to health care workers after percutaneous injury is 0.3-0.4% and after exposure to mucous membranes is 0.09% [1]. The risk for HBV is reported to be 12% but can be a high as 30% in health care workers with a positive HBeAg. The risk for HCV was estimated to be about 3% [9].

A multicenter trial was conducted in France by the Study Group on Hygiene Practices in Interventional Radiology on the potential of HBC exposure in the IR population. Seventy seven radiologists from 11 IR wards participated, and 44% reported one incident of mucous membrane exposure and 52% reported at least one percutaneous injury [6]. This study concluded that compliance with standard precautions, especially with the use of protective clothes and safety material was poor. It also concluded that the risk of HCV transmission from contact with contaminated blood after percutaneous injury ranged from 0.013-0.030 %. This figure was derived simply by multiplying three numbers: an interventional radiologist’s estimated risk of accidental blood exposure, prevalence of HCV in patient population presenting for IR procedures, and the risk of seroconversion after a percutaneous injury.

Baffoy-Fayard *et al.* [6] used a seroconversion rate of 1.8-3.0% based on previously published reports and the calculated a low end risk after percutaneous injury of 1/1,330 whereas the high end risk was found to be 1/800. In the 2003 issue of the Journal of Interventional Radiology (JVIR), a commentary on this particular study was written by MV Marx. She calculated the risk of percutaneous injury to an interventional radiologist using a different set of figures, assuming a worst case scenario. Her numerical conclusions derived a risk of 1/100, which puts a whole new light on the situation. It indicates a 1% chance of contracting hepatitis C due to occupational exposure in an IR practice [7]. These figures are not to be taken lightly, and are only meant to enlighten us about the real threat of blood borne exposure in interventional radiology.

## REGULATIONS ON INFECTION CONTROL

In 1992, the Bloodborne Pathogens Standard was developed by the Occupational Safety and Health Administration (OSHA) and enacted into law in the United States. The Needlestick and Prevention Act (P.L. 106-430) formulated in 2000 is a revision of the OSHA standard and clarifies the need for employers to select safer needle devices and to involve employees (clinicians directly involved in patient care) in identifying and choosing these devices [7]. The updated standard also requires employers to maintain a log of injuries from contaminated sharps. The Guidelines for Prevention of Intravascular Catheter-Related Infections were developed by the Center for Disease Control and Prevention in 1996 and updated in 2002. These guidelines have served as a backbone in the formation of many infectious diseases guidelines globally [13]. Epic, was established in 1998 in the United Kingdom, and its focus was to provide evidence-based guidelines for infection prevention and control in the National Health Service (NHS).

There have been various organisations across the world with evidence-based guidelines on infectious diseases and control, and some of them are listed in Table 1 [13].

**Table 1:** Organisations with evidence-based guidelines on infection control

Center for Disease Control (CDC) www.cdc.gov	Guidelines on prevention of infection and immunisations
Society for Healthcare Epidemiology of America (SHEA) www.shea-online.org	Guidelines on infection control
Epic – Department of Health (NHS) www.epic.tvu.ac.uk	Guidelines for preventing health-care associated infections
British Thoracic Society (BTS) www.brit-thoracic.org.uk	Guidelines on chest infections
British Society for Antimicrobial Chemotherapy (BSAC) www.bsac.org.uk	Guidelines for prophylaxis and treatment of MRSA
Public Health Agency of Canada www.phac.aspc.gc.ca	Guidelines on prevention of infection and immunisations
National Health and Medical Research Council of Australia (NHMRC) www.health.gov.au	Guidelines on infection control
Occupational Safety and Health Administration (OSHA) www.osha.gov	Guidelines on occupational safety and prevention of sharps injuries
Canadian Medical Association (CMA) Infobase www.cma.ca	Guidelines on infectious disease

We will now look at some of the general guidelines and summarize the important elements to be observed by radiology personnel performing interventional procedures (Table 2).

**Table 2:** Summary of guidelines to be observed in interventional radiology suite

**Departmental Responsibilities**	1. Vaccinations Hepatitis B vaccination provided free by employer 2. Infection control plan and training of personnel
**Procedure Safety and Precautions**	1. Proper handling and disposal of sharps – no recapping of needles – avoid passing sharps from one operator to another during procedure – specific containers for sharps disposal – suture with needle holder 2. Safety devices and proper garments – proper hand-washing technique – gloves to be changed after 90 min – gown changed in lengthy and bloody procedures – face shields and eyewear 3. Equipment protection and precaution – Image intensifier and controls at table should be covered with sterile cover – foot pedals covered with non-sterile cover – IR suite mopped after each procedure
**Antibiotic Prophylaxis**	Refer to Table 3
**Post Exposure Recommendations**	Post exposure prophylaxis (PEP) for HBV and HIV No PEP for HCV

## DEPARTMENTAL RESPONSIBILITIES

### 1. Vaccinations

Currently only Hepatitis B is preventable through vaccination and not blood-borne pathogens like HIV and HCV. All health care workers should have Hepatitis B vaccination as a prerequisite to employment. For those not vaccinated, the Hepatitis B vaccination should be provided free by the employer [14].

### 2. Planning and Training

An infection control plan must be developed by employers of the establishment, in which an IR practice is held. This plan must contain schedules for vaccination, training of personnel, post-exposure treatment and also must detail the measures taken by the employer to minimize exposure to health care personnel.

It is recommended that training in infection control, should be provided within 10 days of an employee being hired and annually thereafter. This training should be documented and records of this should be maintained [1].

## PROCEDURE SAFETY AND PRECAUTIONS

### 1. Proper handling and disposal of sharps

Avoid recapping of needles or resheathing of scalpel blades. If this is unavoidable, a one-handed technique is used, where the cap is scooped up by the sharp edge of the instrument or a two-handed technique, where the cap is held by a forceps or any other instrument but not by the operator’s fingers.

Any sharp instrument that is reused should be placed sharp-end down on a sterile pad, plastic container or any other holding device, and placed in a corner of the tray where it will not be readily knocked over. In IR procedures, reassembly of multi-part sharp needles and devices is common and this should be done in the same manner as recapping needles i.e., with the assistance of an instrument and with caution [1,9,14].

Communication of team members is vital during a procedure, especially if there is passing of sharps from one person to another. Generally, this is to be avoided and the “no touch” method should be used, in which the sharp instrument is set down on a stable surface by one team member and then picked up by the second team member after the first has withdrawn his or her hand [9].

Communication with the patient is also important to avoid sudden surprise movements which may lead to injury with sharp objects or instruments. The patient should be informed about the procedure at all times.

Designated containers should be available for all sharps after use. These containers should have a wide opening and should be placed at strategic points in the interventional suite. All team members should be aware of the location of sharps disposal containers. These containers should be emptied when ½ or 2/3 full to prevent injury to the next person using the container and to house keeping staff [14].

Suturing should be performed with needle holders only and never by grasping the needle in one’s fingers. Trying to locate a needle tip by palpation should never be done. Cut the suture and dispose of needle once it is no longer needed. If an injury occurs, the sharp instrument should be discarded immediately [9].

### 2. Safety devices and protective garments

Universal precautions should be strictly adhered to in IR procedures. Proper hand washing techniques with a germicidal agent before putting on protective gloves, and after removal of gloves should be practiced. Glove use during procedures is generally accepted and due to the increasing frequency of occult glove perforations, changing of gloves after 90 min is advised. Gloves should also be changed in the event of a contamination or if perforation is suspected [1,9].

A very common violation is the failure to remove gloves before touching a door knob, telephone or other equipment. This will lead to exposure of the next person who touches that object with an ungloved hand, and should therefore be avoided.

Due to the fact that permeability of fabric is increased after prolonged contact with blood, changing of gowns during lengthy and bloody procedures is advised. Protective face shields and eyewear should be worn whenever there is potential for splashing or spraying of blood or other infectious fluid [14].

### 3. Equipment protection and precaution

Image intensifiers and controls which are connected to the table should be covered with sterile covers to maintain a sterile environment. Similarly, the foot pedals should be covered with a non-sterile cover to preserve disinfection [14].

Parts of the angiographic machine contaminated with blood and other body fluids should be cleaned with a bactericidal agent before the next procedure to prevent cross-contamination. The angiographic suite should be mopped with a germicidal agent after each procedure.

While flushing syringes with saline during a procedure, some blood still remains in the syringe. Ideally, flushing should be done into a closed one-way flow system to prevent spilling or splashing. Glass syringes should never be used unless plastic syringes are not suitable. Luer-lock fittings are preferred over slip fittings for syringes, connecting tubes and drainage systems to prevent the possibility of spillage. If possible, bloodless arterial and venous access puncture systems should be used at the discretion of the operator [1].

## ANTIBIOTIC PROPHYLAXIS

Antibiotic prophylaxis is defined as the use of antibiotics prior to or during a procedure to minimize the risk of infection. There are many disputes regarding the use of prophylactic antibiotics; however, there are some procedures where antibiotics are routinely used universally. According to Ryan *et al.* [15], the benefits of antibiotic prophylaxis in IR practice has never been scientifically proven, as no randomized trials have been carried out. However, antibiotic prophylaxis has been the standard practice in some IR procedures and the use of broad spectrum antibiotics has led to an increasing problem of antibiotic resistance [14, 15]. Stopping the use of antibiotic prophylaxis may combat this problem but is very difficult since prophylactic antibiotic treatment has been a standard part of interventional procedures and many clinicians are worried about legal issues if their patients develop a post-procedural infection.

In choosing a prophylactic antibiotic, the source and pathogen should be considered and the antibiotic should target specific organisms. This will avoid the excessive use of broad spectrum antibiotics. The timing of prophylaxis is also important and it is suggested that prophylactic antibiotics should be given just before or less than two hours before the start of a procedure. An antibiotic administered more than three hours before a procedure increases the incidence of infection five-fold [14]. There are some clinical conditions in which antibiotic cover is mandatory and they include patients with bacterial endocarditis and also biliary and urinary tract interventions in patients with sepsis.

In Table 3we have listed a summary of IR procedures where antibiotic prophylaxis is routinely used.

**Table 3:** Interventional procedures in which antibiotics are routinely used

Uterine fibroid embolisation Hepatic chemoembolisation Splenic embolisation Renal embolisation TIPSS Biliary drainage and biliary intervention Percutaneous nephrostomy and genitourinary procedures Radiofrequency ablation Stent placements through previously placed vascular sheath or hematoma site

## POST EXPOSURE RECOMMENDATIONS

If splashing of contaminated fluid on skin occurs, the exposed skin should be washed immediately with soap and water. If contamination occurs to the mucous membrane, it should be flushed with water and saline.

In the event of a sharps injury with exposure to blood borne pathogens, it should be reported to the relevant authorities as soon as possible and counselling should be given with regards to post exposure prophylaxis (PEP) against HBV and HIV. No effective PEP is available for HCV [9,14].

If the HIV/HBV status of the source is not known, then consent for blood testing should be obtained and the test carried out immediately. If the source is HIV positive, the exposed individual should undergo blood testing at three and six months after exposure if the initial test was negative. During this period of six months, safe sexual practices should be observed and pregnancy and blood donation should be avoided.

According to a CDC report, the risk of infection to HIV was reduced by 79% by the use of ziduvudine (AZT) after percutaneous exposure. Current recommendations call for combination therapy with zidovudine, lamivudine (3TC) and indinavir [9]. However, if the source patients have been on antiretroviral therapy, these recommendations need to be adjusted in view of potential HIV drug resistance.

If the source is HBV positive, PEP consists of the Hepatitis B vaccine in combination with Hepatitis B immune globulin (HBIG). The vaccine should be started within seven days of exposure and HBIG should be administered within 24 hours. This is done if the exposed person has not been vaccinated before or his anti-HBV antibody level is <10mIU/ml after vaccination. If the exposed person has been vaccinated and his anti-HBV antibody level is >10mIU/ml, no treatment is necessary.

Further testing should be done in 4-6 months [1,9]. Counselling is important to alleviate anxiety and fear and to provide advice regarding safe sexual practices and pregnancy.

## CONCLUSION

The risk of blood borne exposure in IR practice is real and from the literature available, it is obvious that there is insufficient knowledge about it. There have not been many studies done regarding the transmission of infection in the interventional radiology suite and more data is required. The worldwide guidelines for infection control are important and are useful in planning rules and regulations in a particular establishment. In general, radiology departments are units that have been unnoticed in terms of hospital infection, but with the advent of interventional procedures, more attention should be given to address the issue of infection control in the interventional suite. There is no doubt that the future will bring more information, data and research on this issue.
